# Supplementary material for: Hemodialysis and Peritoneal Dialysis in Germany from a Health Economic View—A Propensity Score Matched Analysis
Source: Int J Environ Res Public Health. 2022 Oct 27;19(21):14007. doi: 10.3390/ijerph192114007 (PMC9653857; doi:10.3390/ijerph192114007)

Supplementary materials

Table S1. Diagnoses for CKD requiring dialysis

EBM code	Service	Operationalisation
40823	In-center HD, ≥ 18 years	Identification of HD
40824	In-center HD, ≥ 18 years, ≤ 2x per week	Identification of HD
40825	PD, HHD or IPD, ≥ 18 years	Identification of PD, HHD, or IPD
40826	PD, HHD, IPD, ≥ 18 years, ≤ 3 dialysis days per week	Identification of PD, HHD, or IPD
40827	IPD or HHD, ≥ 18 years, ≤ 2x per week	Identification of HHD or IPD
40837	Addition to 40825 for IPD	Differentiation of IPD patients from PD/HHD/IPD
40838	Addition to 40827 for IPD	Differentiation of IPD patients from PD/HHD/IPD

EBM Einheitlicher Bewertungsmaßstab, HD hemodialysis, HHD home hemodialysis, IPD intermittent peritoneal dialysis, PD peritoneal dialysis.

Table S2. Absolute contraindications

Disease group	ICD-10-Codes
Chronic inflammatory bowel disease	K50, K51, K52.8
Diverticulitis of the bowel	K57
Somnolence, sopor and coma	R40
Other disorders of peritoneum	K66

Table S3. Definition of regions

Region	Federal states
North	Bremen, Hamburg, Lower Saxony, Schleswig-Holstein
East	Berlin, Brandenburg, Mecklenburg-Western Pomerania, Saxony, Saxony-Anhalt, Thuringia
South	Baden-Württemberg, Bavaria
West	Hesse, North Rhine-Westphalia, Rhineland-Palatinate, Saarland

Table S4. Charlson Comorbidity Index conditions and weights

**Condition**	**Weight**
AIDS/HIV	6
Any malignancy, including lymphoma and leukemia, except malignant neoplasm of skin	2
Cerebrovascular disease	1
Chronic pulmonary disease	1
Congestive heart failure	1
Dementia	1
Diabetes with chronic complication	2
Diabetes without chronic complication	1
Hemiplegia or paraplegia	2
Metastatic solid tumor	6
Mild liver disease	1
Moderate or severe liver disease	3
Myocardial infarction	1
Peptic ulcer disease	1
Peripheral vascular disease	1
Renal disease	2
Rheumatic disease	1

Table S5. Annual costs per patient (in €) according to type of dialysis (HD or PD) (unmatched dataset; HD: n=6829; PD: n=439)

Cost types	HD	PD	HD-PD[a]	95% CI*	P-value
Total	**48,761**	**46,133**	**2628**	-949–5938	0.130
Outpatient services[1]	23,857	23,332	525	-1037–1908	0.481
Drug expenses	7485	5945	1540	424–2421	0.004
Sick pay	168	579	-411	-619–-221	0.003
Therapeutic aids	731	425	306	164–424	0.003
Inpatient services[1]	16,164	15,710	454	-2654–3042	0.752
Rehabilitation	355	142	213	119–303	0.001

*Confidence intervals were calculated by bootstrapping (n=10,000), [a]Difference of HD and PD. [1]Including costs for dialysis therapy.

Table S6. Poisson regressions (dependent variable: hospitalisations)

Dialysis modality	Variables	Beta	SE	P-value	RR
HD	Women	-0.015	0.047	0.747	0.985
	North	-0.114	0.069	0.101	0.893
	East	-0.079	0.072	0.271	0.924
	South	-0.073	0.054	0.172	0.929
	< 65 years	-0.050	0.049	0.305	0.951
	CCI	0.072	0.007	0.000	1.074
PD	Women	-0.121	0.049	0.013	0.886
	North	-0.065	0.070	0.350	0.937
	East	-0.171	0.077	0.026	0.843
	South	0.043	0.053	0.419	1.044
	< 65 years	-0.024	0.050	0.636	0.977
	CCI	0.076	0.007	0.000	1.079

Figure S1. Overall survival (unmatched dataset; HD: n=6829, PD: n=439, p log-rank = 0.000)

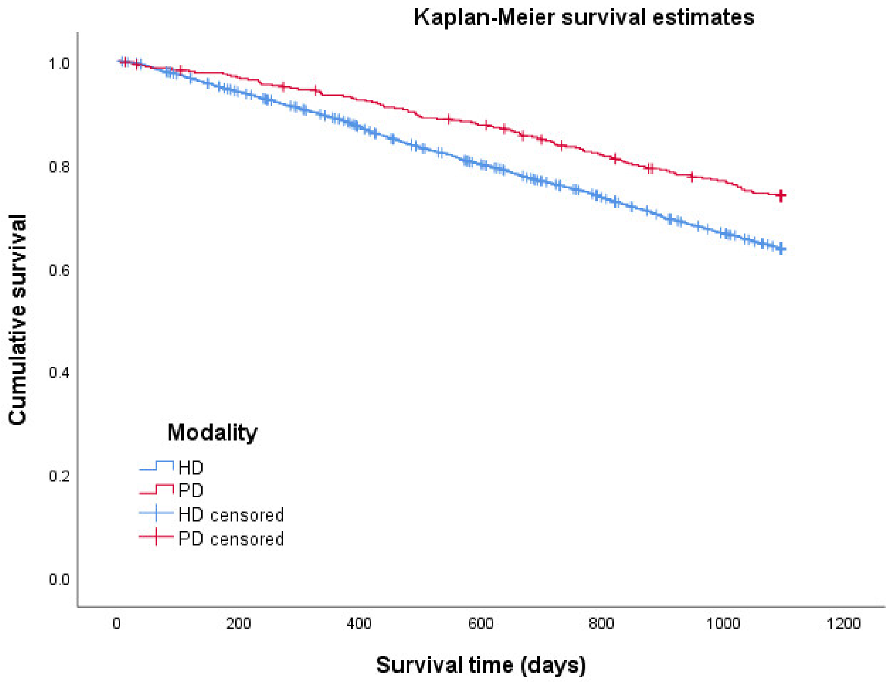

Supplement: Supplementary file 1 [file ijerph-19-14007-s001.zip › ijerph-1923664-supplementary.pdf]
